# Depression and serum 25-hydroxyvitamin D in older adults living at northern latitudes – AGES-Reykjavik Study

**DOI:** 10.1017/jns.2015.27

**Published:** 2015-11-20

**Authors:** Cindy M. Imai, Thorhallur I. Halldorsson, Gudny Eiriksdottir, Mary F. Cotch, Laufey Steingrimsdottir, Inga Thorsdottir, Lenore J. Launer, Tamara Harris, Vilmundur Gudnason, Ingibjorg Gunnarsdottir

**Affiliations:** 1 Unit for Nutrition Research, Landspitali – The National University Hospital of Iceland and Faculty of Food Science and Nutrition, School of Health Sciences, University of Iceland, Eiriksgata 29, 101 Reykjavik, Iceland; 2 Department of Epidemiology Research, Centre for Fetal Programming, Statens Serum Institut, 5, Artillerivej, 2300 Copenhagen S, Denmark; 3 Icelandic Heart Association, Holtasmari 1, 201 Kopavogur, Iceland; 4 Division of Epidemiology and Clinical Applications, National Eye Institute, 10 Center Drive, MSC 1204, Bethesda, MD 20892-1204, USA; 5 National Institute on Aging, Laboratory of Epidemiology, and Population Sciences, 7201 Wisconsin Avenue, Bethesda, MD 20892-9205, USA; 6 Faculty of Medicine, School of Health Sciences, University of Iceland, Vatnsmyrarvegur 16, 101 Reykjavik, Iceland

**Keywords:** Vitamin D, Depression, Older adults, Cross-sectional analyses, Nutritional epidemiology, 25(OH)D, 25-hydroxyvitamin D, AGES-Reykjavik, Age, Gene/Environment Susceptibility–Reykjavik, GDS, Geriatric Depression Scale, MINI, Mini-International Neuropsychiatric Interview

## Abstract

Low vitamin D status may be associated with depression. Few studies have examined vitamin D and depression in older adults living at northern latitudes. The present study cross-sectionally investigated serum 25-hydroxyvitamin D (25(OH)D) status and depression among 5006 community-dwelling older persons (66–96 years) living in Iceland (latitudes 64–66°N). Depressive symptoms were measured by the fifteen-item Geriatric Depression Scale (GDS-15). Current major depressive disorder was assessed according to Diagnostic and Statistical Manual of Mental Disorders, fourth edition (DSM-IV) criteria. Serum 25(OH)D was analysed using chemiluminescence immunoassay and categorised into three groups: deficient (<30 nmol/l); inadequate (30–49·9 nmol/l); and adequate (≥50 nmol/l). There were twenty-eight (2 %) men and fifty (1 %) women with current major depressive disorder. Mean GDS-15 scores for men and women with adequate vitamin D concentrations were 2·1 and 2·2, respectively. Men and women with deficient *v.* adequate vitamin D status had more depressive symptoms (higher GDS-15 scores) (difference 0·7 (95 % CI 0·4, 0·9) and 0·4 (95 % CI 0·1, 0·6), respectively). Furthermore, men with deficient vitamin D status were more likely to have current major depressive disorder (adjusted OR 2·51; 95 % CI 1·03, 6·13) compared with men with adequate vitamin D status. Associations among women were not significant. In this older population living at northern latitudes, deficient vitamin D status may be associated with depression. Further investigations are warranted to evaluate the pathways that may be associated with risk of depression among older adults.

Depression is one of the most common mental disorders and is recognised as a major contributor to the global burden of disease^(^^1^^,^^2^^)^. Individuals of advanced age, in particular, may be prone to developing depression due to life changes such as chronic illness, moving into a retirement facility, and loss of a spouse or close friends^(^^3^^)^. Depressive symptoms are prevalent in approximately 15 % of community-dwelling older persons^(^^4^^)^, with major depressive disorder prevalent in 1–5 % of adults 65 years and older^(^^5^^)^. However, a more recent systematic review estimated the prevalence of major depression in late life (>75 years) to range from 4·6 to 9·3 %^(^^6^^)^, suggesting it may be an increasing problem in old age. Depression risk among older adults may be further exacerbated by an interaction of factors beyond age such as poor dietary intake and time spent indoors which can negatively affect vitamin D status^(^^7^^)^.

The influence of vitamin D on the risk of depression has gathered attention due to the identification of vitamin D receptors in locations of the brain linked to depression^(^^8^^)^. Associations between serum 25-hydroxyvitamin D (25(OH)D) levels and depressive symptoms have primarily been investigated in observational studies and findings have been inconsistent^(^^9^^–^^16^^)^. A meta-analysis of cross-sectional studies reported a non-significant but increased risk of depression with vitamin D status below *v.* above 50 nmol/l (hazard ratio 1·31; 95 % CI 1·00, 1·71); limiting the analyses to participants aged 65 years and older did not change the risk estimates^(^^17^^)^.

Few studies have investigated vitamin D status and depression in older adults living at northern latitudes. In Iceland, where the latitude reaches from 64–66°N, dermal production of vitamin D is limited from October to April^(^^18^^)^. Therefore, intake of a vitamin D-containing supplement is recommended, and cod liver oil has been a traditional component of the Icelandic diet for many decades^(^^19^^)^. The aim of this present study was to investigate associations between vitamin D status measured by serum 25(OH)D concentrations and depression in older adults with data on health and lifestyle factors, and where a combination of cod liver oil intake and unique geographical location results in large variations in serum vitamin D levels.

## Experimental methods

### Study population

This is a cross-sectional analysis of the Age, Gene/Environment Susceptibility–Reykjavik (AGES-Reykjavik) Study; a continuation of the population-based Reykjavik Study initiated by the Icelandic Heart Association^(^^20^^)^. Participants were 5764 older men and women, of mean age 77·0 (sd 5·9) years, living in the greater Reykjavik area who were randomly chosen from survivors who were enrolled in the Reykjavik Study and who agreed to participate in the AGES-Reykjavik Study. Detailed data on medical and lifestyle history were collected. The Mini-International Neuropsychiatric Interview (MINI) was used to assess psychiatric disorders, including major depressive disorder. Participants who exhibited cognitive impairment by scoring <21 on the Mini-Mental State Examination (MMSE) or who were unable to complete the MMSE (*n* 396) were not eligible to take the MINI and were thus excluded from this analysis. Furthermore, individuals with missing serum 25(OH)D levels (*n* 121) and covariates used in the statistical analyses (*n* 241) were excluded, leaving 5006 (43 % males) individuals in the current analysis.

The AGES-Reykjavik Study was approved by the Icelandic National Bioethics Committee (VSN: 00-063) and the MedStar IRB for the Intramural Research Program, Baltimore, MD, USA. Informed written consent was obtained from all participants.

### Measurement of serum 25-hydroxyvitamin D

Fasting blood samples were collected between September 2002 and January 2006 and kept frozen at –80°C until analysis. Measurement of total serum 25(OH)D, including both vitamins D_2_ and D_3_, was processed on-site at the Icelandic Heart Association by means of a direct, competitive chemiluminescence immunoassay using the LIAISON 25 OH Vitamin D total assay (Diasorin, Inc.), hereafter referred to as vitamin D. The interassay CV was <6·5 % when using a frozen serum pool as a control sample and <12·7 % when using Liaison quality controls.

### Cod liver oil intake

Information on intake of cod liver oil was gathered using a FFQ designed for the AGES-Reykjavik Study. Participants were asked how often they were taking cod liver oil currently as well as when they were middle aged (40–50 years). The response categories for cod liver oil intake were: never; less than once per week; 1–2 times per week; 3–4 times per week; 5–6 times per week; or daily. The cod liver oil intake assessed by the FFQ has been shown to be well correlated with quantities collected at midlife when compared with dietary data collected as part of a national dietary survey and intake in old age where the AGES FFQ was compared with 3-d weighed food records completed by the same individuals (Spearman's *ρ* = 0·53 and 0·56, *P* < 0·001 for men and women, respectively, at midlife and *ρ* = 0·51 and 0·42, *P* < 0·001 for men and women, respectively, in old age)^(^^21^^,^^22^^)^.

### Depression

The protocol for diagnosing subjects with depression in the AGES-Reykjavik Study has been previously described^(^^23^^,^^24^^)^. Major depressive disorder was assessed according to Diagnostic and Statistical Manual of Mental Disorders, fourth edition (DSM-IV) criteria using the MINI, a structured diagnostic test designed for epidemiological studies which provides information about past and current (preceding 2 weeks) depressive episodes. Participants completed the MINI if they screened positive for depressive symptoms, i.e. ≥6 on the fifteen-item Geriatric Depression Scale (GDS-15), had occurrences of anxiety, were told by a doctor they had depression, reported ever using antidepressant medications, or if they were using antidepressant medications as evidenced from medication bottles brought to the study interview. All participants completed the GDS-15 which provides self-reported information on depressive symptoms in the preceding week and has been validated in the Icelandic population^(^^25^^)^.

### Covariates

The following covariates were selected based on a potential relationship with serum vitamin D status or depression and could confound the association between them. Age, education (primary, secondary, college, or university), season of blood draw (winter: December–February, spring: March–May, summer: June–August, autumn: September–November), BMI, alcohol intake (<25, 25–50, ≥50 g/week), current smoking (yes/no), marital status (single/divorced/widowed or married/living together), current antidepressant use (yes/no), physical activity (moderate to vigorous <0·05, 0·05–5·0, >5 h/week), multivitamin use (yes/no), and leisure indoor activities (d/month) were assessed by questionnaire or clinical assessment. Diagnosis of type 2 diabetes was based on fasting serum glucose of ≥7 mmol/l, self-reported doctor's diagnosis, and/or use of diabetes medication. Diagnosis of CHD was confirmed by electrocardiography and/or self-reported doctor's diagnosis. Hypertension was diagnosed by having physiological measurements of systolic blood pressure ≥140 mm Hg and/or diastolic blood pressure ≥90 mm Hg, self-reported doctor's diagnosis and/or use of hypertension medications.

### Statistical analyses

Participant characteristics were described using mean and standard deviation for normally distributed variables, median and interquartile range for skewed variables or proportion (%) for dichotomous variables. Men and women were analysed separately because of sex-specific differences in the prevalence of depression^(^^26^^)^. Vitamin D as determined by 25(OH)D concentrations was categorised into three groups: deficient (<30 nmol/l); inadequate (30–49·9 nmol/l); and adequate (≥50 nmol/l) based on recommended levels in the literature^(^^27^^)^. For continuous variables, differences between the three vitamin D categories were identified using one-way ANOVA for normally distributed variables and the Kruskal–Wallis test for non-normally distributed variables. For dichotomous variables, differences between groups were identified with the Pearson χ^2^ test.

The main outcome measures used were depressive symptoms based on the GDS-15 scores and current major depressive disorder based on the MINI. Multivariate linear regression analyses were used to assess the association between vitamin D and GDS-15 scores. Analyses for GDS-15 scores were adjusted for all covariates including antidepressant use. Since the MINI takes into consideration antidepressant medication use, antidepressant use was not adjusted for in the analyses with current major depressive disorder. Logistic regression analyses were used to estimate the OR and 95 % CI of current major depressive disorder with adjustments for the covariates previously described excluding antidepressant use. Adequate vitamin D was the reference category. Analyses were performed using SAS, version 9.2 (SAS Institute). Level of significance was set at *P* ≤ 0·05, two-sided.

## Results

Overall, there were twenty-eight men (2 %) and fifty (1 %) women with current major depressive disorder. Half of the women and 41 % of men in the cohort had deficient or inadequate vitamin D levels. Tables 1 and 2 summarise the participant characteristics by serum vitamin D level separately for men and women. The prevalence of current major depressive disorder was higher among men with deficient (<30 nmol/l) *v.* adequate (≥50 nmol/l) serum vitamin D levels (3 *v.* 1 %) but not among women (1 *v.* 2 %). Both men and women with the lowest vitamin D levels were more likely to have a higher BMI, smoke, live alone, consume less alcohol and cod liver oil, not use multivitamins and were less likely to be physically active compared with those with higher vitamin D status. Women with lower vitamin D status were more likely to use antidepressant medication. Higher GDS-15 scores (i.e. more depressive symptoms) were also observed in both men and women with the lowest vitamin D categories.

**Table 1.** Baseline characteristics of the men by serum 25-hydroxyvitamin D (25(OH)D) status (Mean values and standard deviations, or percentages)

	25(OH)D (nmol/l)						
	Deficient (<30) (*n* 295)		Inadequate (30–49·9) (*n* 574)		Adequate (≥50) (*n* 1261)		
	Mean	sd	Mean	sd	Mean	sd	*P*
Characteristic							
Age (years)	76·2	5·7	76·6	5·5	76·5	5·2	0·58
Height (cm)	175·4	6·2	175·2	6·2	175·8	6·1	0·16
Weight (kg)	83·9	14·8	84·3	13·8	82·2	12·8	0·0038
BMI (kg/m^2^)	27·2	4·3	27·4	3·9	26·6	3·6	<0·0001
Vitamin D (nmol/l)*	27·0	5·5	41·0	10·2	68·6	22·4	<0·0001
GDS-15 score	2·9	2·7	2·3	1·8	2·1	1·8	<0·0001
Current major depressive disorder (%)	3		1		1		0·002
Lifestyle							
Education (%)							0·0117
Primary	17		18		13		
Secondary	54		51		54		
College	15		13		13		
University	14		18		20		
Current smoker (%)	22		12		9		<0·0001
Cod liver oil intake (%)							<0·0001
<Once per week	61		38		16		
1–6 times per week	11		15		9		
Daily	28		47		75		
Antidepressant use (%)	3		2		1		0·06
Multivitamin use (%)	15		21		30		<0·0001
Physical activity (h/week)	0·82	1·96	1·32	2·32	1·69	2·52	<0·0001
Alcohol (g/week)*	4·8	24·1	6·4	26·4	8·0	24·8	0·0098
Leisure activities (d/month)	4·5	3·4	4·6	3·7	4·6	3·5	0·83
Living with partner (%)	66		74		81		<0·0001
Chronic diseases							
Type 2 diabetes (%)	20		17		13		0·0129
CHD (%)	38		33		32		0·18
Hypertension (%)	87		80		79		0·0084

GDS, Geriatric Depression Scale.

* Median and interquartile range.

**Table 2.** Baseline characteristics of the women by 25-hydroxyvitamin D (25(OH)D) status (Mean values and standard deviations, or percentages)

	25(OH)D (nmol/l)						
	Deficient (<30) (*n* 536)		Inadequate (30–49·9) (*n* 912)		Adequate (≥50) (*n* 1428)		
	Mean	sd	Mean	sd	Mean	sd	*P*
Characteristic							
Age (years)	76·4	5·5	76·4	5·7	76·1	5·5	0·47
Height (cm)	160·4	5·7	160·9	5·5	161·2	5·8	0·02
Weight (kg)	73·0	15·0	72·0	13·3	68·8	11·9	<0·0001
BMI (kg/m^2^)	28·3	5·4	27·8	4·8	26·5	4·3	<0·0001
Vitamin D (nmol/l)*	21·8	8·9	40·7	10·1	65·1	19·0	<0·0001
GDS-15 score	2·9	2·2	2·3	2·1	2·2	2·0	<0·0001
Current major depressive disorder (%)	1		2		2		0·14
Lifestyle							
Education (%)							<0·0001
Primary	35		28		26		
Secondary	48		48		48		
College	13		17		20		
University	4		7		6		
Current smoker (%)	20		13		11		<0·0001
Cod liver oil use (%)							<0·0001
<Once per week	57		34		18		
1–6 times per week	9		11		6		
Daily	34		55		76		
Antidepressant use (%)	7		4		5		0·03
Multivitamin use (%)	17		34		40		<0·0001
Physical activity (h/week)	0·60	1·61	1·01	1·99	1·21	2·15	<0·0001
Alcohol (g/week)*	1·6	3·2	1·6	8·0	1·6	13·2	<0·0001
Leisure activities (d/month)	5·2	3·5	5·1	3·4	5·4	3·5	0·16
Living with partner (%)	42		46		50		0·0020
Chronic diseases							
Type 2 diabetes (%)	13		10		7		0·0003
CHD (%)	18		13		13		0·0140
Hypertension (%)	83		82		80		0·33

GDS, Geriatric Depression Scale.

* Median and interquartile range.

### Depression and vitamin D status

Mean GDS-15 scores were 2·1 (sd 1·8) and 2·2 (sd 2·0) for men and women, respectively, with adequate 25(OH)D concentrations. Men and women with deficient *v.* adequate vitamin D status had higher GDS-15 scores (more depressive symptoms) (difference 0·7 (95 % CI 0·4, 0·9) and 0·4 (95 % CI 0·1, 0·6), respectively; Table 3). Findings remained largely unchanged after further adjustment for cod liver oil intake. Men who had deficient vitamin D status were also more likely to be currently depressed even after adjustments for age, season of blood draw and education (OR 3·60; 95 % CI 1·34, 6·95) (Table 4, model 1). The association among men attenuated slightly after adjustments for lifestyle factors and chronic diseases, but remained significant after full multivariate adjustment (OR 2·51; 95 % CI 1·03, 6·13) (Table 4, model 3). Further adjustment for current cod liver oil intake did not markedly change the OR for men (2·67; 95 % CI 1·02, 7·01). There was no significant association between vitamin D status and depression in women.

**Table 3.** Adjusted difference in fifteen-item Geriatric Depression Scale (GDS-15) score compared with men and women with adequate serum 25-hydroxyvitamin D (25(OH)D) status (≥50 nmol/l) (Differences and 95 % confidence intervals)

	Men		Women	
Serum 25(OH)D status (nmol/l)	Difference*†	95 % CI	Difference*†	95 % CI
Deficient (<30)	0·7	0·4, 0·9	0·4	0·1, 0·6
Inadequate (30–49·9)	0·1	−0·1, 0·3	−0·0	−0·2, 0·2
Adequate (≥50)	Reference		Reference	

* Adjusted difference in GDS-15 score compared with adequate serum 25(OH)D.

† Adjusted for season, education, age, BMI, smoking, alcohol intake, antidepressant use, partner status, multivitamin use, physical activity, hypertension, CHD and diabetes.

**Table 4.** Risk of current major depressive disorder by serum 25-hydroxyvitamin D (25(OH)D) level in men and women (Odds ratios and 95 % confidence intervals)

25(OH)D (nmol/l)	Current major depressive disorder (cases/*n*)	Model 1*		Model 2†		Model 3‡	
		OR	95 % CI	OR	95 % CI	OR	95 % CI
Men							
Deficient (<30)	10/285	3·06	1·34, 6·95	2·56	1·05, 6·20	2·51	1·03, 6·13
Inadequate (30–49·9)	3/571	0·45	0·13, 1·58	0·44	0·12, 1·55	0·43	0·12, 1·54
Adequate (≥50)	15/1246	Reference		Reference		Reference	
Women							
Deficient (<30)	13/523	1·26	0·64, 2·47	0·83	0·40, 1·72	0·80	0·39, 1·66
Inadequate (30–49·9)	10/902	0·58	0·28, 1·21	0·46	0·22, 0·97	0·45	0·21, 1·00
Adequate (≥50)	27/1401	Reference		Reference		Reference	

* Adjusted for age, season and education.

† Adjusted for age, season, education, BMI, smoking status, alcohol intake, partner status, multivitamin use and physical activity.

‡ Adjusted for age, season, education, BMI, smoking status, alcohol intake, partner status, multivitamin use, physical activity, type 2 diabetes, CHD and hypertension.

As the presence of chronic disease may influence risk of depression, we ran analyses excluding participants with chronic disease (i.e. hypertension, CHD and diabetes) to determine whether they were driving the association with depression. The adjusted difference between vitamin D status (deficient *v.* adequate) and GDS-15 scores remained significant (difference 1·7 (95 % CI 0·6, 2·8) and 0·7 (95 % CI 0·1, 1·4) for men and women, respectively). Results for current major depressive disorder and vitamin D were not significant among men and women with no chronic disease (combined due to the low number of depression cases).

## Discussion

In this older population-based cohort living at northern latitudes, we found a modest inverse association between vitamin D status and depressive symptoms among both men and women. Furthermore, older men with deficient serum vitamin D levels (<30 nmol/l) were twice as likely to be currently depressed compared with comparably aged men with adequate vitamin D levels (≥50 nmol/l) even after accounting for other factors such as diabetes and hypertension which might confer a health-related reason for depression. We found no association between vitamin D status and current major depressive disorder among women.

Previous cross-sectional studies linking low serum 25(OH)D levels with depressive symptoms have reported stronger inverse associations among women compared with men^(^^10^^,^^16^^,^^28^^)^, while findings from the Concord Health and Ageing in Men (CHAMP) study and European Male Ageing Study (EMAS) reported significant inverse associations among men^(^^12^^,^^29^^)^. Two large population-based studies in China and the USA reported no association between depressive symptoms and serum 25(OH)D levels but sex-specific results were not provided^(^^14^^,^^15^^)^. There have been varying results from prospective studies. In a Italian study of older adults, women with serum 25(OH)D levels below 50 nmol/l were at greater risk of developing a depressed mood after 6 years (hazard ratio 2·09; 95 % CI 1·25, 3·49)^(^^28^^)^. Another study of elderly Chinese men reported an inverse association between vitamin D status and depression at baseline but not at follow-up 4 years later^(^^30^^)^.

Physical activity, if performed outdoors, is important for both dermal production of vitamin D and its potential to alleviate depressive symptoms^(^^31^^)^. Less physical activity was associated with reduced vitamin D levels and it modestly attenuated the OR for depression when we adjusted for it in our analyses. Individuals with depression also have a greater likelihood of staying indoors and having poorer dietary habits, thereby affecting vitamin D status^(^^7^^)^. However, the frequency of leisure indoor activities was not significantly different among the vitamin D categories in both men and women. Season may also influence vitamin D levels and we found vitamin D levels to be, on average, 5 nmol/l higher in the summer months compared with the winter months for both men and women (data not shown). However, we did not find seasonal differences on the association between vitamin D concentrations and depression in our cohort. Reasons for these small differences may be partially attributed to the northern latitude and limited amount of sun exposure. During the summer months in Iceland, people tend to still wear long sleeves and trousers, and the high frequency of cod liver oil use throughout the year may negate some seasonal variations.

The significant relationship that we observed between vitamin D status and depression among men is contrary to prior cross-sectional reports where analyses were performed separately by sex^(^^10^^,^^16^^)^. There is some evidence that men place more value on autonomy^(^^32^^)^ and an increase in dependency, particularly secondary to chronic diseases, has been associated with depression^(^^33^^)^. The presence of diabetes, in particular, can double the odds of depression^(^^34^^)^. The men and women in our cohort with deficient vitamin D status, compared with higher levels, had the highest prevalence of type 2 diabetes and other chronic diseases; however, adjustment for these diseases in our analyses did not markedly change the findings. Our sensitivity analyses excluding participants with chronic diseases also yielded significant results. It is possible that lifestyle changes were connected to depressive status, although we were unable with the data available to differentiate whether this might be the case. We did observe that cod liver oil intake was inversely correlated with vitamin D status, and the mean difference in serum 25(OH)D concentrations between those with no *v.* daily intake was around 20 nmol/l. Further adjustments for current cod liver oil intake in our analyses of vitamin D status and depression did not change the significant findings and other factors not accounted for by cod liver oil intake are likely to be of importance. However, as cod liver oil intake and vitamin D status are closely connected, it is difficult to separate completely the effects of these two variables.

A possible explanation for the lack of association among women with current major depressive disorder may be that following menopause, the primary predictor of depression is prior depressive history^(^^35^^)^ although certain life events^(^^3^^)^, as previously stated, can affect depression risk. However, the inverse association with current depressive symptoms suggests that vitamin D may still be an important nutrient with respect to protection against pathways that could lead to depression^(^^36^^)^. The sex-specific differences we observed suggest that future studies should analyse men and women separately, especially as our combined analyses of data on men and women comparing deficiency *v.* adequate vitamin D status yielded a null result (OR for current major depressive disorder 1·30; 95 % CI 0·75, 2·27).

Our findings in men and those from previous observational studies^(^^10^^,^^12^^,^^16^^,^^28^^,^^29^^)^ suggest that higher vitamin D levels may potentially be associated with less depression. However, reports from intervention studies have been mixed. Three large randomised controlled trials found no association between vitamin D supplementation and depression among postmenopausal women^(^^37^^–^^39^^)^. In a randomised controlled trial in Norway, a high dose of vitamin D provided to men and women aged 30–75 years with low (mean 40·1 nmol/l) serum 25(OH)D levels for 6 months did not improve depression scores compared with controls who had taken a placebo^(^^40^^)^. A separate Norwegian study reported positive effects of vitamin D supplementation on reducing depressive symptoms among 441 overweight or obese individuals, although few participants exhibited depressive symptoms at the onset^(^^41^^)^. The inconsistencies between the studies could be attributed to variations in the participants’ baseline vitamin D status, intake and sun exposure particularly at northern latitudes.

It is also possible that vitamin D deficiency (serum levels <30 nmol/l) but not inadequacy (30–49·9 nmol/l) is associated with depression. Researchers from the Australian CHAMP study suggest that the optimal 25(OH)D range in older men to be between 50 and 74·9 nmol/l^(^^12^^)^. However, in a country at more northern latitudes such as Iceland, maintaining vitamin D status above deficient levels may be sufficient^(^^42^^,^^43^^)^.

### Strengths and limitations

The strength of the AGES-Reykjavik Study lies in the extensive data gathered in this population-based cohort allowing for adjustments of potential confounders such as various lifestyle behaviours and chronic diseases. Although there was a high prevalence of elderly with deficient (<30 nmol/l) serum vitamin D levels, reflecting the advanced age and living conditions (northern latitude), some of the cohort benefited to some degree by the traditional practice of ingesting cod liver oil on a regular basis.

There are several limitations. The cross-sectional nature of our study and the single measure of vitamin D status leaves the possibility for reverse causality where older individuals with a past history of depression may have changed their dietary habits and this could be reflected in their serum vitamin D status as measured by 25(OH)D concentration. However, there is some evidence from a Norwegian study that vitamin D status tracks longitudinally over time^(^^44^^)^. In this cohort, cod liver oil intake during midlife (40–50 years of age) collected from the FFQ and which has been validated^(^^22^^)^ was strongly correlated with cod liver oil intake in late life, suggesting that the frequency of use for this vitamin D-containing supplement is indicative of long-term use. In addition, our results indicated that there was minimal seasonal variation in vitamin D levels, suggesting that the serum vitamin D levels in this cohort are likely to reflect longstanding vitamin D status. We do acknowledge that vitamin D intake and status can be markers of a healthier lifestyle and the presence of depression or depressive symptoms may alter behaviours affecting vitamin D status such as sun exposure. The lifetime prevalence of major depressive disorder in Iceland is between 15 and 25 %, with a point prevalence of 3·8–4·8 %^(^^45^^)^. The relatively lower prevalence of current depression in this cohort and the simultaneous frequent use of cod liver oil may limit the generalisability of our results. We also acknowledge that the strength of the association between vitamin D and GDS-15 scores, as well as with major depressive disorder, is small and further research in needed. Longitudinal assessment of both depression and vitamin D values would provide greater insight into how these two measures are related and whether the temporal relationship differs over time in men compared with women.

### Conclusion

Our findings contribute to the growing evidence supporting the association between vitamin D status and mental health. It is important to note that the association between depression and vitamin D in men was observed at a relatively low level of serum 25(OH)D (<30 nmol/l). In northern latitudes, maintaining vitamin D levels above 30 nmol/l may protect older adults against adverse health conditions which are associated with depression. Future research on the effects of vitamin D should examine in-depth differences between men and women when assessing the risk of depression among older adults.
